# Antiprotozoal Activity of Triazole Derivatives of Dehydroabietic Acid and Oleanolic Acid

**DOI:** 10.3390/molecules22030369

**Published:** 2017-02-28

**Authors:** Mariano Walter Pertino, Celeste Vega, Miriam Rolón, Cathia Coronel, Antonieta Rojas de Arias, Guillermo Schmeda-Hirschmann

**Affiliations:** 1 Laboratorio de Química de Productos Naturales, Instituto de Química de Recursos Naturales, Universidad de Talca, 3460000 Talca, Chile; schmeda@utalca.cl; 2 Centro para el Desarrollo de la Investigación Científica (CEDIC), Manduvirá 635 entre 15 de Agosto y O’Leary, Barrio La Encarnación 1255, 2511 Asunción, Paraguay; mcvegagomez@gmail.com (C.V.); rolonmiriam@gmail.com (M.R.); cathiacoronel@gmail.com (C.C.); rojasdearias@gmail.com (A.R.d.A.)

**Keywords:** cytotoxicity, dehydroabietic acid, *Leishmania* spp., oleanolic acid, triazole derivatives, *Trypanosoma cruzi*

## Abstract

Tropical parasitic diseases such as Chagas disease and leishmaniasis are considered a major public health problem affecting hundreds of millions of people worldwide. As the drugs currently used to treat these diseases have several disadvantages and side effects, there is an urgent need for new drugs with better selectivity and less toxicity. Structural modifications of naturally occurring and synthetic compounds using click chemistry have enabled access to derivatives with promising antiparasitic activity. The antiprotozoal activity of the terpenes dehydroabietic acid, dehydroabietinol, oleanolic acid, and 34 synthetic derivatives were evaluated against epimastigote forms of *Trypanosoma cruzi* and promastigotes of *Leishmaniabraziliensis* and *Leishmania infantum*. The cytotoxicity of the compounds was assessed on NCTC-Clone 929 cells. The activity of the compounds was moderate and the antiparasitic effect was associated with the linker length between the diterpene and the triazole in dehydroabietinol derivatives. For the oleanolic acid derivatives, a free carboxylic acid function led to better antiparasitic activity.

## 1. Introduction

Chagas is endemic in 21 countries in Latin America. It is a zoonotic parasitosis considered one of the most important neglected diseases in Latin America with a total of 520,043 DALYs (years of disability-adjusted life) [1,2,3,4]. Currently, it was estimated that 90–100 million people are at risk of contracting Chagas disease and eight million are infected by the flagellate protozoan *Trypanosoma cruzi* with approximately 12,000 deaths and 56,000 new cases per year [5]. An estimated 5.4 million people will develop cardiac Chagas disease [1,6], while 900,000 will have megaesophagus and megacolon [1]. Given the globalization of our days, the disease exists in at least 10 non-endemic countries. Currently, more than two million people infected with Chagas disease live in Spain and other European countries [7], and even in other continents [8].

There are two phases of Chagas disease. The first acute phase last around four months and is characterized by large numbers of parasites circulating in the patient blood. However, just 5% of patients are detected during the acute phase and can be cured [9]. In the chronic phase, the parasitemia is low, and the pathognomonic manifestation could appear as chronic cardiomyopathy, megas in the digestive tract, as well as damage to the central nervous system. Two major groups of the *T. cruzi* parasite strains have been described elsewhere (TcI and TcII). TcI is associated with the sylvatic cycle of transmission while TcII is associated with the domestic cycle [10,11]. It was observed that a specific geographical distribution of these two major groups. TcI strains predominate in the Amazon basin and the northern countries of South America, Central America, and Mexico, while TcII is commonly found in the southern part of South America. The megas are usually found in the south. By this fact, TcII strains are incriminated as more pathogenic than TcI strains [11].

Chances of being cured in the chronic phase, by conventional treatments, are very low. This disease is considered, together with leishmaniasis and African Trypanosomiasis, an extremely neglected disease. In the case of the poorest countries in the developing world, they are characterized by the fact that they affect poor populations in developing countries, which do not add services, being excluded socioeconomically and, consequently, pharmaceutical markets [12].

Leishmaniasis is generally endemic in 98 countries and territories in the world, and is responsible for a disease burden of 2.35 million DALYs, 2.3% of which occur in the Americas (50,668) [4]. More than 12 million people are infected with leishmaniasis, and 350 million are at risk. It is estimated that 75% of all cases of cutaneous leishmaniasis are concentrated in 10 countries, four of which are in the Americas: Brazil, Colombia, Peru, and Nicaragua. Brazil is one of the six countries where 90% of the cases of visceral leishmaniasis have been found; the others are Ethiopia, India, Bangladesh, Sudan, and South Sudan. In the Americas, an average of 60,000 cases of cutaneous and mucosal leishmaniasis and 4000 cases of visceral leishmaniasis are diagnosed each year, with a mortality rate of 7% [13,14].

One of the most widely distributed species in Latin America is *Leishmania braziliensis* that can produce both cutaneous and mucocutaneous leishmaniasis. Another important species is *L. infantum* that can produce visceral leishmaniasis, the most common species identified in animals especially dogs [15]. These two species were selected to perform the in vitro leishmanicidal activity of the compounds tested in this study. The life cycle of both parasites spends an important development inside the reservoir or in the human body. The trypomastigote forms of *T. cruzi* enter the organism through the skin or mucous membranes, then pass into the cells of the endothelial reticulum or into the circulatory or lymphatic torrent [16,17]. When the parasite enters the cells, it is transformed into amastigotes, which multiply until it completely invades the cell and becomes epimastigotes. Afterwards, the cell in the trypomastigotes is broken, and the trypomastigotes are released into the circulatory torrent. At this moment, the trypomastigotes can be detected by a fresh examination, and they later invade new tissues and become amastigotes, thus closing the cycle. A similar cycle is observed in *Leishmania* spp. Promastigotes are phagocytized by macrophages and other types of mononuclear phagocytic cells, and transformation allows them to reach amastigotes, the tissue stage of the parasite, which multiply by simple division and proceed to infect other mononuclear phagocytic cells [18,19].

The promastigotes and epimastigotes are the extracellular dividing forms of the parasite inside the insect vectors of *Leishmania* spp. and *T. cruzi*, respectively. As they are easy to cultivate under laboratory conditions, these models are a good choice for preliminary in vitro screening [20,21].

Despite the progress in the chemotherapy of the mentioned parasitic diseases, there is an urgent need for new drugs. Although Chagas disease can be treated with drugs such as nifurtimox and benznidazole, there are reports on which these treatments fail [22]. The drugs currently used by leishmaniasis have discreet effectiveness, long treatment times, high cost, and adverse effects, and the parasites rapidly develop resistance to drugs [23]. Therefore, the discovery of new drugs is a priority. Just 3.8% of drugs approved between 2000 and 2011 were for neglected diseases such as leishmaniasis, Chagas disease, and sleeping sickness, which together represent more than 10% of the global burden of diseases [24].

Several natural products of plants such as phenolic, alkaloids, saponins, and terpenes show potent and selective bioactivity for the treatment of tropical diseases [25]. Abietane-type diterpenes isolated from roots of *Salvia cilicica* (Lamiaceae), and triterpenes such as ursolic acid and betulinaldehyde, display significant in vitro antileishmanial activity. Recently, different research groups have used the Cu(I) catalyzed 1,3-dipolar-cycloaddition reaction of azide and alkyne (CuAAC) to generated 1,2,3-triazoles on the search for antiparasitic drugs. Using this reaction, they obtained triazol-derivatives of sugars, benznidazoles, naphthoquinones, and sterols with excellent antiparasitic properties [26,27,28,29,30]. In previous work, we reported the synthesis and antiproliferative activity of dehydroabietic acid (DHA) coupled with 1,2,3-triazoles (**1**–**16**) and 1,2,3-triazole-substituted oleanolic acid (OA) derivatives (**17**–**34**). Most of the compounds showed low antiproliferative activity on tumor and normal cells. Therefore, they should be regarded as compounds with low toxicity, which can be an advantage when looking for selective drugs [20]. In the present work, compounds **1**–**34** were examined for a trypanocidal effect on *T. cruzi* and in vitro activity against two promastigote stages of *Leishmania* spp.

## 2. Results and Discussion

**Figure 1:**
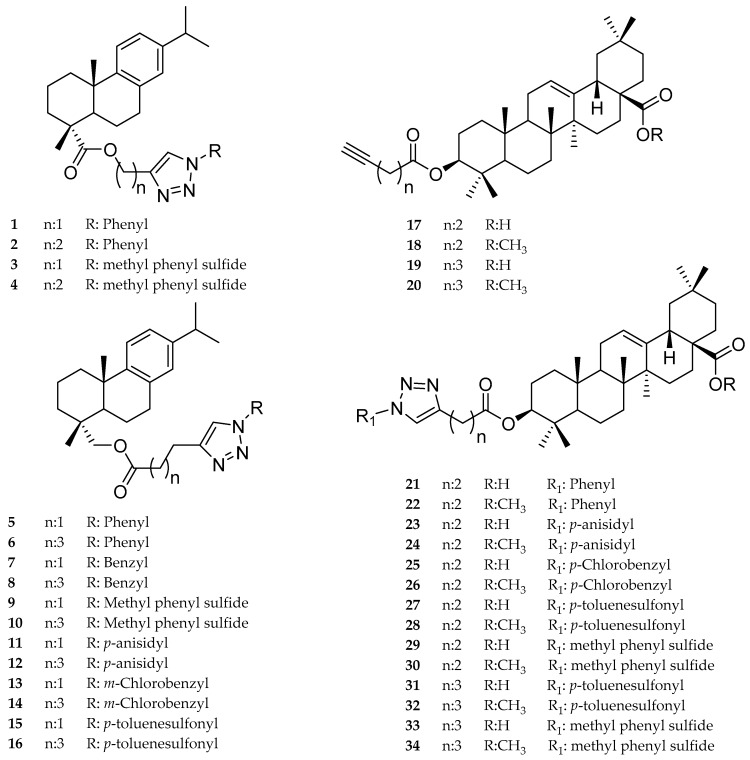
The structure of compounds **1**–**34** synthesized and evaluated as antiprotozoal agents in vitro.

From the starting terpenes OA, DHA, and dehydroabietinol, 34 synthetic derivatives were prepared (Figure 1) and assessed against epimastigote forms of *T. cruzi*, promastigotes from *Leishmania* spp. as well as for cytotoxicity. The results are summarized in Table 1.

### 2.1. Dehydroabietic Acid Derivatives 

The activity on *T. cruzi* of DHA, dehydroabietinol, and compounds **1**–**16** was moderate. When comparing with DHA, the effect was stronger for dehydroabietinol, with IC_50_ of 66 µg/mL compared with 212 µg/mL for DHA. The most active products were the derivatives **1**, **4**, and **15** with IC_50_ values of 46, 61, and 69 µg/mL, respectively. Compound **1** has a CH_2_ linker between the diterpene and the triazole, while **2** (IC_50_ > 256 µg/mL) bears two CH_2_ units in the linker. However, when the triazole presents a methyl phenyl sulfide (compounds **3** and **4**), the effect is stronger for the compound with two CH_2_ units in the linker (>256 vs. 61 µg/mL). The same trend was observed for compounds **15** and **16**, with IC_50_ values of 69 and 199 µg/mL, respectively. The most active product **15** shows two CH_2_ units in the linker while **16** presents four CH_2_ units in the linker, respectively. These results are showing the importance of linker length in the activity against *T. cruzi* in this type of compounds. Subsequent studies including linkers of different lengths would be necessary to identify the ideal chain length to obtain derivatives with potential activity against *T. cruzi*. Inhibition of *Leishmania* proliferation in vitro was tested against two promastigote strains. DHA, dehydroabietinol, and their derivatives, **1**, **4**, **15**, and **16**, were the most active compounds against both species evaluated with IC_50_ values of 44, 63, 53, 64, 89, and 71 µg/mL against *L. braziliensis* and 72, 40, 60, 64, 53, and 73 µg/mL against *L. infantum*, respectively. These compounds except the derivative **16** were also active against *T. cruzi*. The same structure–activity trend discussed for *T. cruzi* is observed for *Leishmania*. When comparing the evaluated derivatives versus the reference compound (pentamidine, IC_50_: 3.3 μg/mL), a marked difference in activity against *Leishmania* was observed. However, pentamidine has several side effects including nephrotoxicity, so alternative drugs are still needed. The cytotoxicity of the compounds was evaluated on NCTC-Clone 929 fibroblasts. Among the evaluated derivatives only compounds **4**, **15**, **16**, and **17** showed moderate cytotoxic activity with IC_50_ values in the range of 129–232 µg/mL. With the exception of compound **17**, these dehydroabietic derivatives are those that have shown the best antiparasitic activity in this study (**4**, **15**, and **16**), so it could be deduced that the cytotoxicity is relatively non-specific, and the molecules could be acting indistinctly on the cells and the parasites.

Natural abietane diterpenes have shown interesting antiparasitic activities. For example, Samoylenko et al. [31] reported the antileishmanial activity of ferruginol and totarol against *L. donovani* promastigotes with IC_50_ values of 3.5 μg/mL for both compounds and IC_90_ values of 7.0 and 6.9 μg/mL, respectively. Another naturally occurring abietane with antileishmanial activity is 12-methoxycarnosic acid with an IC_50_ value of 0.75 μM against axenically grown *L. donovani* amastigotes. Recently, Olmo et al. [32] reported five abietane diterpenoids as candidates for treating Chagas disease. These compounds were synthesized from abietic acid, essentially all of them being dehydroabietic acid derivatives. In vitro and in vivo activity and the possible mechanism of action of the compounds were studied. In vitro trypanocidal evaluation of the compounds against different forms of the parasite showed better effects than the reference drug. Two compounds were the best candidates, being 29- and 52-fold more active than benznidazole against amastigotes forms. Additionally, they exhibit good activity in vivo and low toxicity.

In recent studies, dehydroabietic acid was treated with different amino acid in search of antiprotozoal activity [33]. The derivatives were evaluated against *L. donovani* amastigotes and *T. cruzi* amastigotes. Several of them showed good activity—in some cases, higher than the reference compound benznidazole. In continuation of this study, the same group reported the preparation of new amides, but this time starting from dehydroabietylamine [34]. The derivatives were evaluated against *L. donovani* and *T. cruzi*. The most promising compound showed IC_50_ values of 0.37 μM and 0.6 μM, with a selectivity index value of 63 and 58 against *L. donovani* axenic amastigotes and *T. cruzi*, respectively. Subsequent to this work, Dea-Ayuela et al. [35] reported new dehydroabietylamine derivatives with antileishmanial activity against promastigotes of *L. donovani*, *L. infantum*, *L. amazonensis*, and *L. guyanensis* with IC_50_ values in the range of 2.2–46.8 μM.

### 2.2. Oleanolic Acid Derivatives

For the OA derivatives (compounds **17**–**34**), only two compounds (**27** and **28**) showed a relevant effect on *T. cruzi* with IC_50_ values of 43 and 61 µg/mL, respectively. They differ in the free (**27**) or methylated COOH function of the triterpene (**28**) but show the same *p*-toluensulfonyl moiety in the triazole ring. On the other hand, the results on leishmanicidal activity show that only compounds **19** and **31** have a slight effect. Compounds **19** presents activity against both *L. braziliensis* and *L. infantum* promastigotes, with IC_50_ values of 109 µg/mL and 155 µg/mL, respectively. Compound **31** showed activity only against *L. infantum* promastigote with an IC_50_ of 128 µg/mL. All other compounds should be regarded as inactive on *Leishmania* promastigotes (IC_50_ > 256 µg/mL).

Several natural pentacyclic triterpenoid such as epi-oleanolic acid, betulinic acid, and betulinaldehyde, among others display leishmanicidal activity against different *Leishmania* species [36,37]. Torres-Santos et al. [38] reported the antileishmanial activity of ursolic acid and oleanolic acid. Ursolic acid showed IC_50_ values of 5 and 27 μg/mL, and oleanolic acid displayed IC_50_ values of 10 and 11 μg/mL against the promastigote and amastigote stage of *L. amazonensis*, respectively. Recently, Melo et al. [39] determined the activity of oleanolic acid against three different *Leishmania* species (*L. braziliensis*, *L. amazonensis*, and *L. infantum*). The reported leishmanicidal activity against promastigotes and amastigote shows IC_50_ values in the range of 30–66 μM and 38–69 μM, respectively. The results differ from our study, where the IC_50_ values of the OA was >256 μg/mL. This difference can be explained by protozoal strains, isolated from different sources and the low solubility in water of OA.

Rodríguez-Hernández et al. [40] reported the synthesis and leishmanicidal activity of hederagenin derivatives prepared by click chemistry. Hederagenin is a triterpene which differs from oleanolic acid by an additional hydroxyl group at C-23. Unlike the OA derivatives reported in this paper, where esters were at the C-3 position, the Hederagenin derivatives were prepared on the carboxylic acid in C-28. Hederagenin esters and amides containing 1,2,3-triazole derivatives were evaluated against amastigote forms of *L. infantum*. The four additional active derivatives showed IC_50_ values of 2 μΜ, 9.7 μΜ, 11 μΜ, and 12 μΜ.

Taking together, the synthetic strategy of coupling a diterpene (DHA) or a triterpene (OA) with a triazole using click chemistry allowed us to obtain structural diversity for structure–activity studies. Some of the derivatives presented better antiprotozoal effects than did the starting compounds, suggesting that further modifications might lead to better antiprotozoal effects for this group of compounds.

## 3. Materials and Methods

### 3.1. Chemistry

Dehydroabietic acid (DHA) was obtained from commercial rosin as described in [41]. Oleanolic acid (OA) was isolated from the aerial parts of *Fabiana imbricata* [42]. The triazole derivatives were prepared as described previously (Figure 1) [41,42]. Briefly, DHA was methylated with CH_2_N_2_ to afford dehydroabietic acid methyl ester. Reduction of the methyl ester with LiAlH_4_ in THF gave the corresponding alcohol (dehydroabietinol). Starting from DHA and its alcohol, 16 abietane-triazole compounds were obtained using click chemistry [41]. The alkyl esters of DHA and its alcohol were prepared using *N*,*N*-dicyclohexylcarbodiimide (DCC)/ dimethylaminopyridine (DMAP) and appropriate alcohol or acid with alkyl function. The alkynyl esters were treated with different azides in *t*-BuOH/H_2_O using CuSO_4_.5H_2_O and sodium ascorbate to obtain the derivatives **1**–**16** (Figure 1).

Oleanolic acid (OA) derivatives contained 1,2,3-triazoles linked to the 3-O function and a free or methylated COOH function at C-28 were prepared as reported [42]. OA was treated with the appropriate alkyne acid/DCC/DMAP to afford the alkynyl esters **17**–**20**. The alkynyl esters and different azides were dissolved in CH_2_Cl_2_/H_2_O (1:1), followed by click chemistry conditions (CuSO_4_·5H_2_O/sodium ascorbate) to afford compounds **21**–**34** (Figure 1).

### 3.2. Antiprotozoal Assays

For in vitro studies of *T. cruzi*, the clone CL-B5 was used (donated by Faculty of Pharmacy, Complutense University, Madrid, Spain). Parasites were stably transfected with the *Escherichia coli* β-galactosidase gene (lacZ). Epimastigotes were grown at 28 °C in liver infusion tryptose broth (Difco, Detroit, MI, USA) with 10% fetal bovine serum (FBS) (Gibco, Carlsbad, CA, USA), penicillin (Ern S.A., Barcelona, Spain), and streptomycin (Reig Jofré S.A., Barcelona, Spain), as described previously [20], and harvested during the exponential growth phase. Briefly, the screening assay was performed in 96-well microplates (Sarstedt, Inc., Newton, NC, USA) with cultures that had not reached the stationary phase. Epimastigotes were seeded at 2 × 10^5^ parasites/mL in 200 µL growth media. The plates were then incubated with the respective compounds at 28 °C for 72 h; afterwards, 50 µL of chlorophenol red-β-d-galactopyranoside (CPRG) solution was added, resulting in a final concentration of 200 µM. The plates were incubated at 37 °C for an additional 4 h and then read at 595 nm. Each concentration was tested in triplicate [21]. The efficacy of each compound was estimated by calculating the IC_50_ values. These values were calculated by the sigmoidal dose–response curve adjustment using the statistical software program Graph-Pad Prims 3.0 (GraphPhad, San Diego, CA, USA). Benznidazole was used as the reference drug.

Culture of *L. braziliensis* (MHOM/BR/75/M2904), and *L. infantum* (MHOM/FR/91/LEM2259V) was obtained from the Facultad de Farmacia (Universidad Complutense de, Madrid, Spain). The maintenance of the strains, the form of cultivation, and the isolation of shape promastigota were performed following the procedures described by Roldos et al. [20]. The promastigotes were grown at 22 °C in Schneider’s *Drosophila* medium supplemented with 20% FBS. The assay was performed using a modification of a previous method [43]. Promastigotes (2 × 10^6^ parasites/well) were cultured in 96-well plastic plates. Compounds were dissolved in dimethylsulfoxide (DMSO). Different dilutions of the compounds with a final volume up to 200 µL were added. After 48 h at 26 °C, 20 µL of a 2 mM resazurin solution was added, and the oxidation-reduction was quantified at 570 and 600 nm. The solution of resazurin was prepared at 2.5 mM in phosphate buffered solution (PBS), pH 7.4, and filtered through 0.22 µm prior to use. All tests were carried out in triplicate. Resazurin sodium salt was obtained from Sigma-Aldrich (St. Louis, MO, USA) and stored at 4 °C protected from light. The efficacy of each compound was estimated by calculating the IC_50_ values.

### 3.3. Cytotoxicity

The cell line used was NCTC-Clone 929, which was grown in Minimal Essential Medium (Sigma) supplemented with 10% heat-inactivated FBS, penicillin G (100 U/mL), and streptomycin (100 µg/mL). Cell cultures were maintained at 37 °C in a humidified 5% CO_2_ atmosphere. The procedure for cell viability measurement was evaluated with resazurin by a colorimetric method. The cells were plated in 96-microtiter plates at 3 × 10^4^ cells per well in a 100 µL growth medium. The cells were grown overnight at 37 °C, 5% CO_2_. Thereafter, the medium was removed and the compounds were added in a 200 µL medium for 24 h. After incubation, 20 µL of a 2 mM resazurin solution was added to each well. The plates were incubated for 3 h to allow optimal oxidation-reduction. The reduction of resazurin was determined by dual wavelength absorbance measurement at 490 and 595 nm. Background was subtracted. Each concentration was assayed three times. Medium and drug controls were used in each test as blanks [44].

## 4. Conclusions

In the present work, 34 terpene-triazole derivatives were synthesized starting from the natural products dehydroabietic acid, dehydroabietinol, and oleanolic acid. The antiprotozoal activity of the compounds was evaluated against epimastigote forms of *T. cruzi*, promastigotes of *L. braziliensis* and *L. infantum*, and cytotoxicity on NCTC-Clone 929 cells. The compounds that showed moderate activity were the most active diterpene DHA, dehydroabietinol, and the derivatives **1**, **4**, **15**, **16**, **27**, and **28**. The antiparasitic effect was associated with the linker length between the diterpene and the triazole in dehydroabietinol derivatives. For the oleanolic acid derivatives, a free carboxylic acid function led to better antiparasitic activity. Further preclinical studies on both protozoa could be carried out on the tissue phase of both parasites (amastigotes), as well as in vivo assays in experimentally infected mice, in order to obtain more information about their potential use of its antiprotozoal activity.

## Figures and Tables

**Table 1:** Antiprotozoal activity of the starting terpenes dehydroabietic acid (DHA), oleanolic acid (OA), and synthetic derivatives **1**–**34** towards *Leishmania* promastigotes, *Trypanosoma cruzi* epimastigotes, and cytotoxicity on NCTC-Clone 929 cells. The results are presented as IC_50_ data in (µg/mL).

Compound	Leishmanicidal Activity on Promastigotes (µg/mL)		Antiepimastigote Activity of *T. cruzi* (µg/mL)	NCTC-Clone 929 Fibroblasts (µg/mL)
	*L. braziliensis*	*L. infantum*	*T. cruzi*	
**DHA**	44	72	212	-
**Dehydroabietinol**	63	40	66	-
**1**	53	60	46	>256
**2**	>256	>256	>256	>256
**3**	>256	>256	>256	>256
**4**	64	64	61	189
**5**	218	193	223	>256
**6**	>256	>256	>256	>256
**7**	>256	>256	>256	>256
**8**	>256	>256	>256	>256
**9**	>256	>256	>256	>256
**10**	>256	>256	>256	>256
**11**	>256	>256	>256	>256
**12**	>256	>256	>256	>256
**13**	242	>256	>256	>256
**14**	>256	>256	>256	>256
**15**	89	53	69	232
**16**	71	73	199	129
**OA**	>256	>256	>256	-
**17**	>256	>256	252	196
**18**	>256	>256	>256	>256
**19**	109	155	>256	>256
**20**	>256	>256	>256	>256
**21**	>256	>256	>256	>256
**22**	>256	>256	>256	>256
**23**	>256	>256	>256	>256
**24**	>256	>256	>256	>256
**25**	>256	>256	>256	>256
**26**	>256	>256	>256	>256
**27**	>256	>256	43	>256
**28**	>256	>256	61	>256
**29**	>256	>256	>256	>256
**30**	>256	>256	>256	>256
**31**	>256	128	>256	>256
**32**	>256	>256	>256	-
**33**	>256	>256	>256	-
**34**	>256	>256	>256	-
**Pentamidine**	3.3	3.3	-	-
**Benzimidazole**	-	-	15.0	-
